# Differentiation of *Escherichia fergusonii* and *Escherichia coli* Isolated from Patients with Inflammatory Bowel Disease/Ischemic Colitis and Their Antimicrobial Susceptibility Patterns

**DOI:** 10.3390/antibiotics12010154

**Published:** 2023-01-11

**Authors:** Ram Hari Dahal, Yoon-Jung Choi, Shukho Kim, Jungmin Kim

**Affiliations:** Department of Microbiology, School of Medicine, Kyungpook National University, Daegu 41944, Republic of Korea

**Keywords:** gut microbiome, gut colonization, inflammatory bowel disease (IBD), ischemic colitis (IC), antimicrobial resistance (AMR), multidrug resistance (MDR), ESBL-producing *E. coli*

## Abstract

Genotypically, 16S rRNA gene sequence analysis clearly differentiates between species. However, species delineation between *Escherichia fergusonii* and *Escherichia coli* is much more difficult and cannot be distinguished by 16S rRNA gene sequences alone. Hence, in this study, we attempted to differentiate *E. fergusonii* and *E. coli* isolated from faecal samples of disease-associated Korean individuals with inflammatory bowel disease (IBD)/ischemic colitis (IC) and test the antimicrobial susceptibility patterns of isolated strains. Phylogenetic analysis was performed using the adenylate kinase (*adk)* housekeeping gene from the *E. coli* multi locus sequence typing (MLST) scheme. Antimicrobial susceptibility and minimum inhibitory concentration (MIC) of all disease-associated strains in addition to healthy control isolates to 14 antibiotics were determined by broth microdilution-based technique. Next, 83 isolates from 11 disease-associated faecal samples were identified as *E. fergusonii* using 16S rRNA gene sequence analysis. Phylogenetic analysis using the *adk* gene from *E. coli* MLST scheme revealed that most of the strains (94%) were *E. coli*. A total of 58 resistance patterns were obtained from 83 strains of disease-associated (IBD/IC) isolates. All isolates were resistant to at least one tested antimicrobial agent, with the highest resistance against erythromycin (88.0%), ampicillin (86.7%), ciprofloxacin (73.5%), cephalothin (72.3%), gentamicin (59%), trimethoprim-sulfamethoxazole (53%), cefotaxime (49.4%), and ceftriaxone (48.2%). A total of 90.7% of isolates were extended-spectrum beta-lactamase (ESBL)-producers among the resistant strains to third-generation cephalosporins (cefotaxime or ceftriaxone). ESBL-producing *E. coli* isolates from patients with Crohn’s disease (CD), ulcerative colitis (UC), and ischemic colitis (IC) were 92.3%, 82.4%, and 100%, respectively. In conclusion, *adk*-based phylogenetic analysis may be the most accurate method for distinguishing *E. coli* and *E. fergusonii* from *Escherichia* genus. We identified four loci in *adk* gene sequences which makes it easier to discriminate between *E. coli* and *E. fergusonii*. Additionally, we believe that gut colonization by multidrug-resistant ESBL-producing *E. coli* may play a significant role in IBD/IC pathogenesis.

## 1. Introduction

Inflammatory bowel disease (IBD) is an intestinal disorder that causes chronic inflammation in the gastrointestinal (GI) tract, including the digestive tract, colon, and rectum. IBD comprises two diseases, namely, Crohn’s disease (CD) and ulcerative colitis (UC), and both affect the GI tract of the digestive system [1]. The exact aetiology of IBD remains unknown. Various studies suggest potential links to genetics, immunological factors such as innate immunity and adaptive immunity, and environmental factors such as geographic and seasonal variability, ethnicity, diet, and lifestyle (medications, anxiety, depressions, cigarette smoking, and alcohols consumption) [2,3,4]. Multiple studies have hypothesized that the gut microbiota is a major factor in pathogenesis of IBD and several microbial species have been suggested to play a role in pathogenicity [5,6]. No specific microbial species has been linked to the pathogenesis of IBD [1]. However, certain microbial species, such as *Clostridioides difficile*, *Klebsiella pneumoniae*, *Yersinia* spp. (*Y. enterocolitica*, *Y. pseudotuberculosis*, and *Y. intermedia*), *Salmonella enterica*, *Proteus mirabilis*, and *Escherichia coli* have been implicated in IBD relapses [6,7,8,9,10,11,12]. Although *E. coli* were associated with IBD and multidrug-resistant *E. coli* strains have been isolated from IBD patients [13,14], it is unknown whether the organism plays a role in early inflammatory relapses or secondary inflammatory processes.

Ischemic colitis (IC) is another type of inflammatory disorder in the large intestine (particularly, inflammation in the colon due to reduced blood flow). Inflammation of the colon, intestinal injury, and necrosis may result from IC [15]. Usually, IC incidence occurs in old age (60 years old or above), whereas IBD has been reported in those aged 20–39 years [15,16]. Similar to IBD, *E. coli* has been linked to the pathogenesis of IC [17]. Both cases (IBD and IC) suggest that *E. coli* strains play a facilitative role in promoting the disease.

The genus *Escherichia* comprises six validly published species, namely, *Escherichia albertii*, *Escherichia coli*, *Escherichia fergusonii*, *Escherichia hermannii*, *Escherichia marmotae*, and *Escherichia ruysiae* (https://lpsn.dsmz.de/genus/escherichia; accessed on 2 January 2023) [18]. *E. coli* was initially identified by Migula as “*Bacillus coli*” in 1895, and Castellani and Chalmers reclassified “*Bacillus coli*” as *Escherichia coli* in 1919 [19,20]. Since then, numerous *E. coli* strains with over 10,000 serotypes have been isolated, characterized, and assigned to various phylotypes [21,22,23]. Since the first *E. coli* genome sequence was published in 1997 [24], >32,687 genome sequences have been deposited in public databases (https://www.ncbi.nlm.nih.gov/genome/?term=Escherichia+coli; accessed on 2 January 2023). *E. fergusonii* was first isolated by Farmer et al. from human blood in 1985 [25]. Since then, strains of *E. fergusonii* have been frequently isolated from human patients with wound infections, bacteremia, urinary tract infections, pancreatic carcinoma, endophthalmitis, pleuritis, and diarrhea [23].

*E. coli* and *E. fergusonii* are the closest relatives within the genus *Escherichia*; they share approximately 64% DNA–DNA hybridization [25], which makes them difficult to discriminate based on 16S rRNA gene sequencing alone. *E. coli* is distinguishable from *E. fergusonii* from its ability to ferment sorbitol and lactose. However, pathogenic *E. coli*, such as the O157:H7 strain, does not ferment sorbitol, making it difficult to discriminate. Moreover, phenotypic identification using an automated instrument, such as the Vitek 2 automated system, may also result in false identification [26]. For accurate discrimination, phylogenetic analysis using housekeeping genes would be the best method.

Antimicrobial resistance (AMR) is a major global health risk owing to the high risk of microbial disease transmission and treatment failure [27]. In 2019, there were an estimated 4.95 million deaths associated with global AMR, and in the same year, the Centers for Disease Control and Prevention (CDC) estimated the cost of AMR to be $55 billion in the United States alone [28,29]. Consequently, AMR not only contributes to the deterioration of human health but also increases patient morbidity and mortality, along with the economic burden. More importantly, colonization of the gut by multidrug-resistant extended-spectrum beta-lactamase (ESBL)-producing *E. coli* may promote IBD/IC [6,30,31]. Tracking AMR and minimizing the spread of multidrug-resistant (MDR) bacteria, in addition to the proper use of antibiotics, are crucial measures to combat antimicrobial resistance, given that patients with IBD or IC are at greater risk owing to their increased exposure to antibiotic treatments [32].

This study aimed to differentiate *E. fergusonii* and *E. coli* isolated from 11 faecal samples of disease-associated South Korean individuals with IBD or IC and to determine the antimicrobial susceptibility of these isolates along with healthy controls.

## 2. Materials and Methods

### 2.1. Patients with IBD/IC and Healthy Controls (HCs)

We obtained 21 faecal samples from the Department of Laboratory Medicine at Kyungpook National University Hospital in Daegu, South Korea, 10 of which were from HCs. The faecal samples used in this study were residual samples used in the diagnostic tests of patients in Laboratory Medicine. Four of the eleven samples were from patients diagnosed with UC, three from those diagnosed with CD, and four from those diagnosed with IC. This study was approved by the institutional review board (IRB) of Kyungpook National University Hospital (KNUH 2021-03-011-002).

### 2.2. Isolation and Preservation of Strains from Disease-Associated Patients and HCs

All isolates (disease-associated, *n* = 83; HCs, *n* = 50) used in this study were isolated from faecal samples of patients with CD (*n* = 30), UC (*n* = 30), IC (*n* = 23), and HCs (*n* = 50) during the study of culturomics to isolate previously uncultivated human gut microbiota associated with IBD or IC (unpublished). Briefly, fresh faecal samples obtained from KNUH were immediately placed in an anaerobic gas pouch (BD GasPak EZ^TM^ Pouch Systems; BD; Franklin Lakes, NJ, USA) and transferred to the laboratory’s anaerobic gas chamber (BACTRON anaerobic chamber; Sheldon Manufacturing, Inc.; Cornelius, OR, USA). One gram of each stool sample was enriched in 9 mL of defibrinated sheep blood and incubated at 37 °C for 2 days under aerobic and anaerobic conditions. Serial dilutions up to 10^−8^ were made in phosphate buffered saline (PBS), and 100 µL of each enriched faecal sample (10^−5^–10^−9^) was plated on 34 uniquely defined agar plates (Table S1, see Supplementary Materials) and incubated at 37 °C for 3–7 days under both conditions (aerobic and anaerobic). Each distinct colony on agar plate was selected and streaked onto a new agar plate until a pure culture was obtained. For long-term preservation, pure colonies were stored in 50% glycerol stock at −80 °C.

### 2.3. Identification & Characterization of Strains

Per the manufacturer’s instructions, bacterial DNA was extracted from faecal samples collected from patients with IBD/IC and healthy individuals using a FastDNA^®^ spin Kit for soil (MP Biomedicals, Santa Ana, CA, USA). PCR was used to amplify the 16S rRNA gene using universal primers (27F and 1492R) [33]. All strains were sequenced using an Applied Biosystems 3770XL DNA analyser with a BigDye Terminator cycle sequencing Kit v.3.1 (Applied Biosystems, Waltham, MA, USA). Almost complete sequences of 16S rRNA genes were assembled with SeqMan software version 7.1.0 (DNASTAR Inc., Madison, WI, USA), and the resulting sequences were deposited in the National Center for Biotechnology Information (NCBI) database (Tables S2 and S3). All strains were identified using 16S rRNA gene sequences by comparing the top-hit sequences of type strains from the EzBioCloud server (https://www.ezbiocloud.net/identify; accessed on 20 November 2022) or nucleotide BLAST from the NCBI database (https://blast.ncbi.nlm.nih.gov/Blast.cgi?PAGE_TYPE=BlastSearch; accessed on 20 November 2022). In addition, phylogenetic analysis using *adk* gene sequences from *E. coli* MLST (multi locus sequence typing) scheme was used to distinguish *E. coli* from *E. fergusonii* [26]. All 16S rRNA and *adk* gene sequences of IBD and IC strains were deposited in the NCBI database (Tables S2 and S4).

### 2.4. PCR Amplification of adk

The PCR primers *adk*F (5′–ATT CTG CTT GGC GCT CCG GG–3′) and *adk*R (5′–CCG TCA ACT TTC GCG TAT TT–3′) were used to amplify the *adk* gene from all 83 disease-associated isolates. *E. coli* ATCC 25922, *E. coli* KCTC 2441^T^, and *E. fergusonii* KCTC 22525^T^ were used as positive controls. Next, oligonucleotide primers were synthesized by Bioneer Inc. (Daejeon, Republic of Korea). PCR amplification was performed in a reacton mixture of 50 μL containing 0.25 μL TaKaRa Taq (5 U/μL), 5 μL 10X PCR buffer (Mg^2+^ plus), 4 μL dNTP mixture (2.5 mM each), 0.2 μM each primer, and 1.5 μL DNA template under the following cycling conditions: 95 °C for 2 min; 30 cycles of 1 min at 95 °C, 1 min at 54 °C, 2 min at 72 °C followed by 5 min at 72 °C. The amplification of 583 bp products was confirmed by performing 1% agarose gel electrophoresis. The PCR products were then purified using the Sol^TM^ gel & PCR purification kit (SolGent Co. Ltd., Daejeon, Republic of Korea) per the manufacturers’ instructions. Sequencing was performed as previously described.

### 2.5. Phylogenetic Analyses

In this study, the 16S rRNA gene sequences of all 83 (disease-associated) isolates, along with *E. coli* and *E. fergusonii* type species were used to conduct phylogenetic analysis. Furthermore, the 16S rRNA gene sequences of *E. coli* O157:H7 str. Sakai, *E. coli* str. K-12 substr. MG1655, and *E. fergusonii* FDAARGOS_1499 were included, with *E. hermanii* CIP 103176^T^ serving as an outgroup. The 16S rRNA gene sequences of these strains were retrieved from the GenBank database and aligned using the sina Aligner (https://www.arb-silva.de/aligner; accessed on 22 November 2022) [34]. The phylogenetic trees were reconstructed using mega version 11 [35]. The phylogenetic trees were reconstructed using the neighbour-joining (NJ) tree making algorithms [36]. The evolutionary distances were calculated using the Kimura 2-parameter model [37], and 1000 replicates were used for the bootstrap analysis [38]. Subsequently, another phylogenetic analysis was performed using the *adk* gene sequences of all 83 disease-associated (IBD/IC) isolates and reference strains as described previously, but with 400 bootstrap replicates, to evaluate the topological accuracy of the tree.

### 2.6. Antimicrobial Susceptibility Testing

Antimicrobial susceptibility testing was performed in Mueller-Hinton broth using the broth microdilution method per Clinical Laboratory Standards Institute (CLSI) guidelines [39] with the following 14 antibiotics: amoxicillin–clavulanic acid (AMC), amikacin (AMK), ampicillin (AMP), ciprofloxacin (CIP), chloramphenicol (CHL), colistin (CST), cephalothin (CEF), ceftriaxone (CRO), cefotaxime (CTX), erythromycin (ERY), gentamicin (GEN), meropenem (MEM), trimethoprim–sulfamethoxazole (SXT), and tetracycline (TET).

Isolates that were resistant to one or more third-generation cephalosporins (CRO or CTX) were tested for the production of ESBL using the double disc synergy test (DDST) with the following antibiotic disks (Sigma): amoxicillin–clavulanic acid (AMC; 20/10 μg), aztreonam (ATM; 30 µg), ceftazidime (CAZ; 30 µg), cefotaxime (CTX; 30 µg), and ceftriaxone (CRO; 30 µg). DDST was performed on a Mueller-Hinton agar plate as recommended by CLSI [39]. AMC (20/10 μg) was placed at the centre of the plate, and the other disks were spaced 15 mm apart. Any distortion or expansion of the zone of inhibition toward the AMC disk was interpreted as the presence of ESBL-producing strain [40]. *Klebsiella pneumoniae* ATCC 700603 and *E. coli* ATCC 25922 were used as positive and negative controls, respectively.

In addition, meropenem-resistant isolates were subjected to a modified Hodge test for carbapenems production [41]. *K. pneumoniae* KNU 1115 and *Pseudomonas aeruginosa* ATCC 27853 were used as positive and negative controls, respectively. Moreover, the production of carbapenemase was confirmed using the RAPIDEC^®^ CARBA NP (bioMérieux) test kit.

## 3. Results

### 3.1. Phylogenetic Analyses

*E. fergusonii* was identified as the top-hit blast of 16S rRNA gene sequences of 83 strains isolated from disease-associated (IBD or IC) faecal samples (Table S2) and 50 strains isolated from HCs (Table S3).

**Figure 1:**
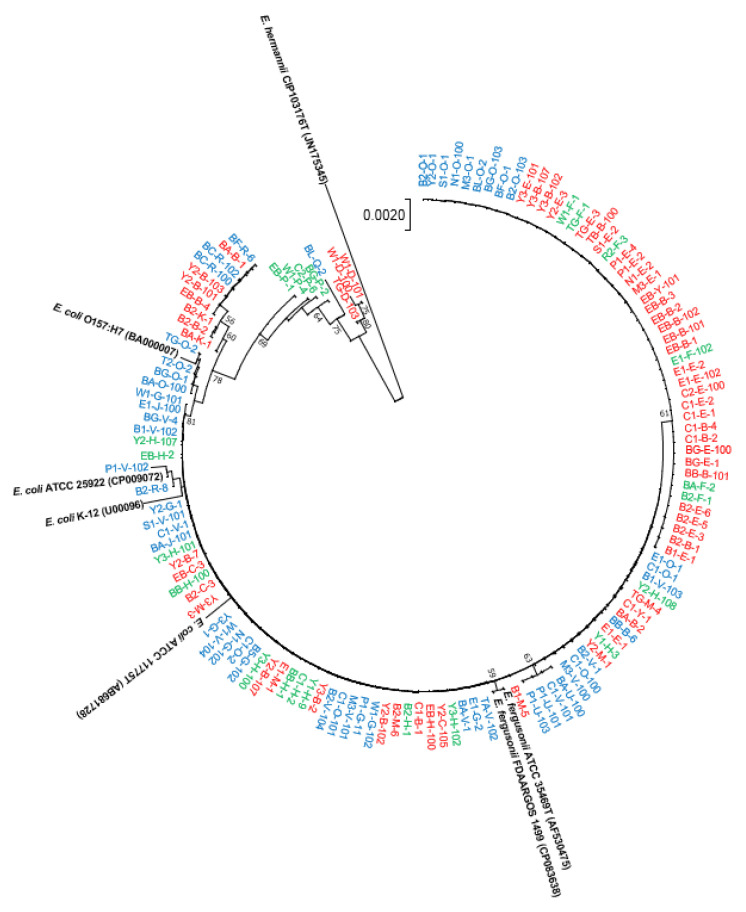
The neighbour-joining phylogenetic tree based on 16S rRNA gene sequences of isolated *E. coli*/*E. fergusonii* strains from patients with IBD or IC patients and healthy controls (HCs). The numbers at the nodes indicate the percentage of 1000 bootstrap replicates, and *Escherichia hermannii* CIP 103176^T^ was used an outgroup. The accession numbers of retrieved 16S rRNA gene sequences are given in parentheses. Strains names with colour codes (red = IBD; green = IC; and blue = HCs).

**Figure 2:**
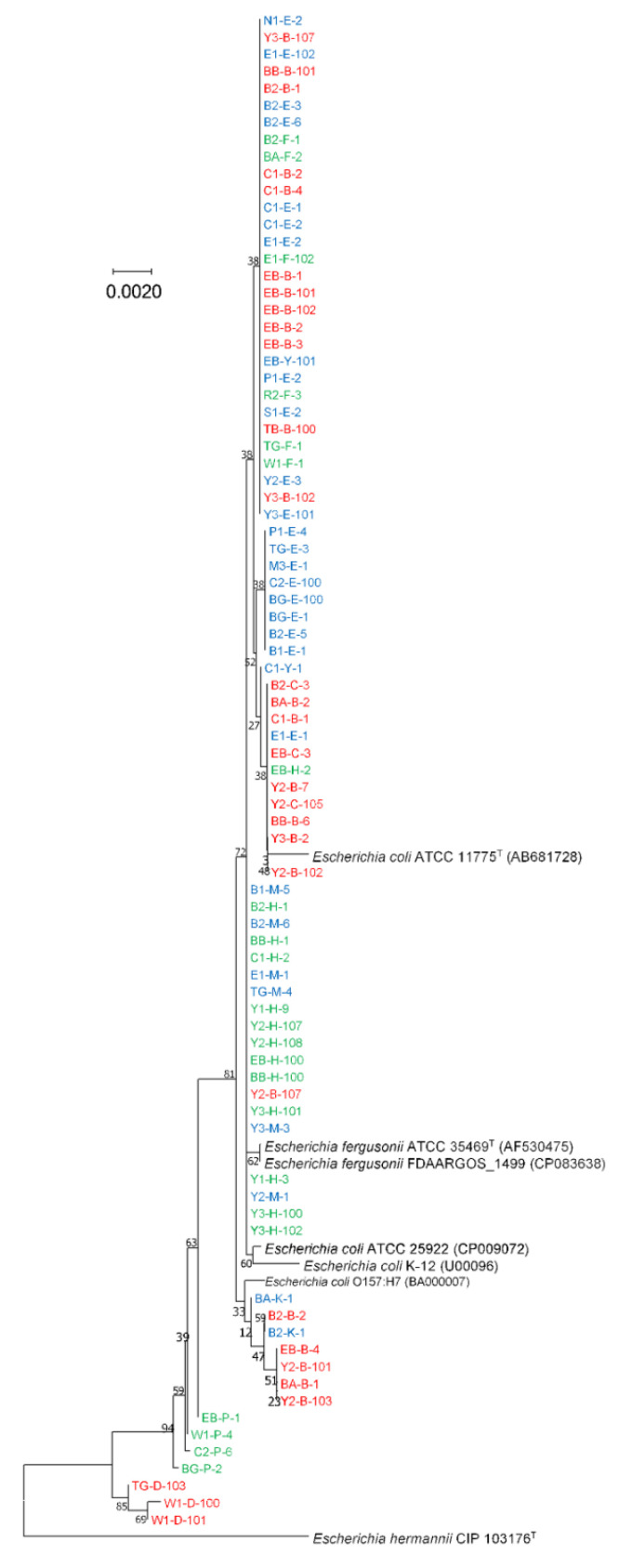
The neighbour-joining phylogenetic tree based on 16S rRNA gene sequences of isolated *E. coli*/*E. fergusonii* strains from patients with IBD or IC. The numbers at the nodes indicate the percentage of 1000 bootstrap replicates. *Escherichia hermannii* CIP 103176T was used an outgroup. The accession numbers of retrieved 16S rRNA gene sequences are given in parentheses. Strain names in red and blue represent IBD (red = CD; and blue = UC), and green represents IC.

The 16S rRNA gene sequences of all isolated strains (IBD/IC, *n* = 83; and HC, *n* = 50) clustered with both *E. coli* type species (*E. coli* ATCC 11775^T^) and *E. fergusonii* type species (*E. fergusonii* ATCC 35469^T^), as well as other *E. coli* or *E. fergusonii* strains (Figure 1). There was no distinct clade to distinguish the isolates either from disease-associated (IBD and IC) or HC faecal samples. Furthermore, we reconstructed the neighbour-joining phylogenetic tree based on the 16S rRNA gene sequences of all isolates (*n* = 83) from disease-associated faecal samples (CD, UC, and IC) using the NJ method (Figure 2). On the phylogenetic tree, all isolates clustered with both *E. coli* type species (*E. coli* ATCC 11775^T^) and *E. fergusonii* type species (*E. fergusonii* ATCC 35469^T^), as well as other *E. coli* or *E. fergusonii* strains (Figure 2). In addition, we also reconstructed the phylogenetic tree based on its 16S rRNA gene sequences with reference sources (HC and IBD; HC and CD; HC and UC; and HC and IC). However, in all phylogenetic trees, isolates could not be distinguished based on reference sources (either from healthy controls or disease-associated faecal samples) (Figures S1–S4, see Supplementary Materials).

Phylogenetic analysis revealed that most isolates clustered with *E. coli* type species, whereas over 15 isolates clustered with *E. fergusonii* type species. However, EB-P-1, W1-P4, C2-P-6, BG-P-2, BG-O-2, TG-D-103, W1-D-100, and W1-D-101 did not cluster with either *E. coli* or *E. fergusonii* (Figure 1 and Figure 2). Hence, phylogenetic analyses of the 16S rRNA gene sequences of *E. coli* and *E. fergusonii* yielded inconclusive results concerning their identification.

**Figure 3:**
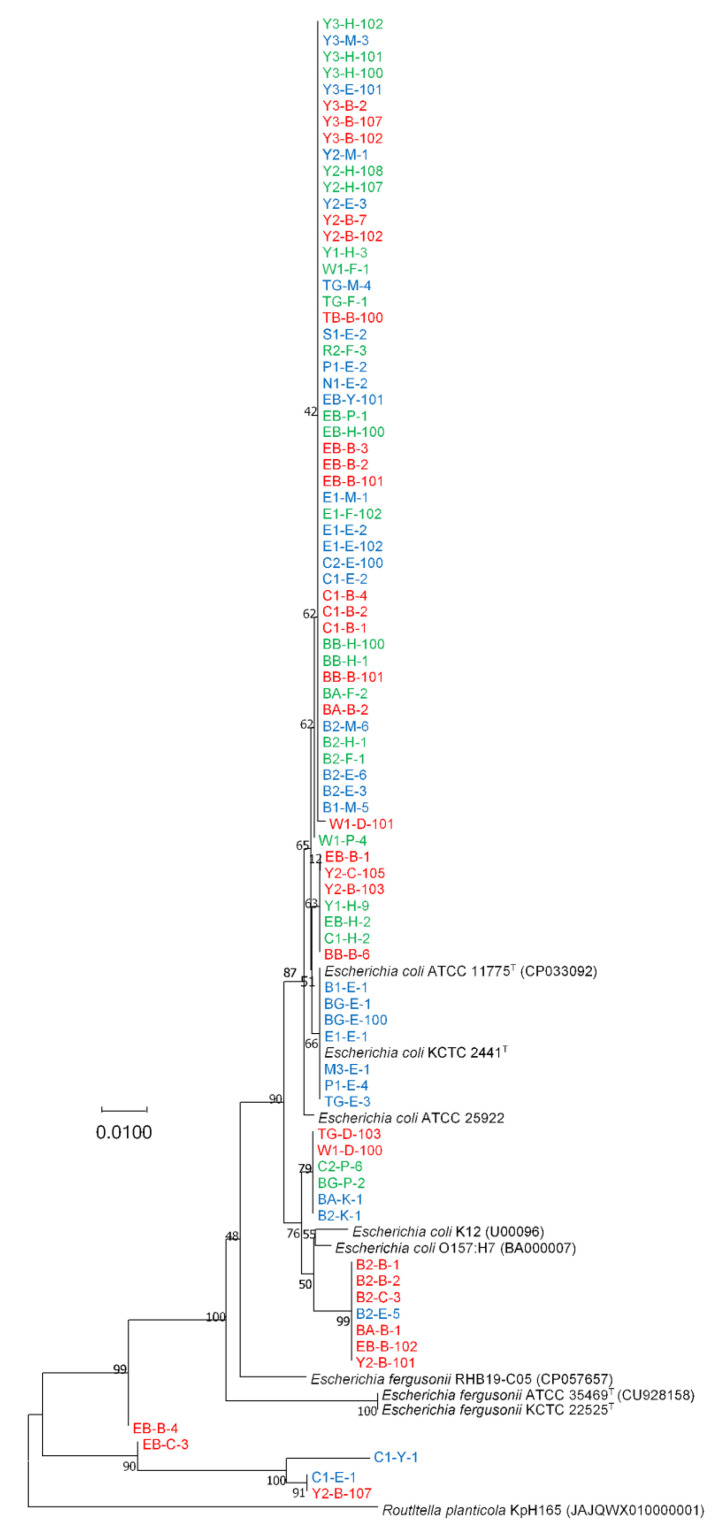
The neighbour-joining phylogenetic tree based on 583-bp *adk* gene sequences of isolated *E. coli* strains from patients with IBD or IC. The numbers at the nodes indicate the percentage of 400 bootstrap replicates. *Routltella planticola* KpH165 was used an outgroup. Strain names in red and blue represent IBD (red = CD; and blue = UC), and green represents IC. The accession numbers are provided in parentheses whose *adk* gene sequences were retrieved from whole genome sequences.

Except for few strains, phylogenetic analysis using the *adk* gene revealed that nearly all isolates (94.0%) belonged to *E. coli* and clustered with 90% bootstrap support (Figure 3). None of the isolates clustered with *E. fergusonii,* and the phylogenetic position of *E. fergusonii* species was distinct from the cluster of *E. coli* (Figure 3). Notably, some strains, including C1-E-1, C1-Y-1, EB-B-4, EB-C-3, and Y2-B-107, were surprisingly distant from both *E. coli* and *E. fergusonii* strains. Based on their respective *adk* gene sequences, these five strains were identified as *Citrobacter freundii* by comparing them with the NCBI standard database using the MegaBLAST and BLASTN methods (Table S4).

### 3.2. Differentiation of E. coli and E. fergusonii Based on adk Gene Sequences

The size of the *adk* gene sequence is 645 bp. We extracted 645 bp *adk* gene sequences of type species and representatives of *E. coli* and *E. fergusonii* species, including *E. coli* ATCC 11775^T^ (CP033092), *E. coli* O157:H7 (BA000007), *E. coli* K12 (U00096), *E. fergusonii* ATCC 35469^T^ (CU928158), and *E. fergusonii* RHB19-C05 (CP057657), from respective whole genome sequences and aligned all the *adk* gene sequences of 83 isolates with *adk* gene sequences from *E. coli* KCTC 2441^T^, *E. coli* 25922, and *E. fergusonii* KCTC 22525^T^ using a multalign interface server [42]. After alignment analysis, we observed four nucleotide differences in *adk* gene sequences between *E. coli* and *E. fergusonii* at position 93, 96, 477, and 549 (Table 1 and Figure S5).

### 3.3. Antimicrobial Susceptibility

**Figure 4:**
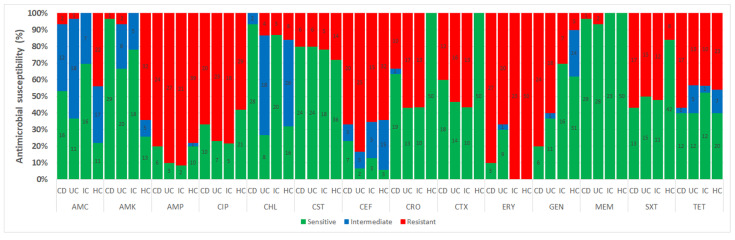
Antimicrobial susceptibility of 83 isolates from faecal samples of patients with CD, UC, and IC and 50 isolates from healthy controls. Number in the bar graph indicate the number of isolates.

**Figure 5:**
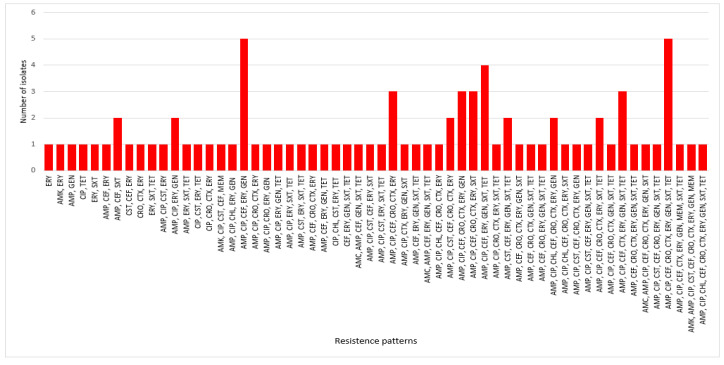
Antimicrobial resistance patterns of 83 isolates from disease-associated faecal samples (IBD/IC).

In this study, 133 isolates (disease-associated, *n* = 83; HCs, *n* = 50) exhibited resistance to at least one antimicrobial agent (Figure 4 and Tables S5 and S6). Approximately 94% of HC isolates and 99% of disease-associated isolates were MDR (Tables S5 and S6). More than 24% of disease-associated isolates displayed the highest MDR patterns against seven antimicrobials, followed by 19.3% against five antimicrobials, and 14.5% against eight MDR patterns (Table S5). Across 83 disease-associated isolates, a total of 58 MDR combination patterns were observed (Figure 5 and Table S5). Two MDR patterns involving five antimicrobials (AMP, CIP, CEF, ERY, and GEN) and eight (AMP, CIP, CEF, CRO, CTX, ERY, GEN, SXT, and TET) antimicrobials were prevalent (6.0%, 5/83 each isolate) in disease-associated isolates (Table S5). Similarly, 41, 27, 22, and 19 MDR combination patterns were observed in the HC, CD, UC, and IC isolates, respectively (Tables S6–S9).

**Figure 6:**
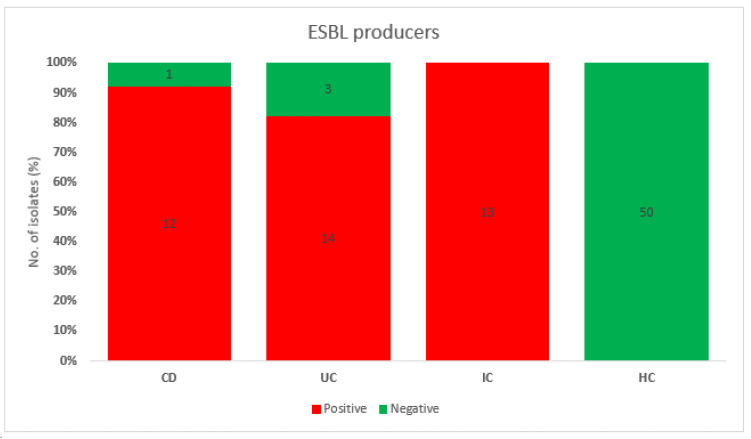
ESBL producing isolates from patients with CD, UC, and IC and the HC group.

For disease-associated faecal samples, ERY (88.0%), AMP (86.7%), CIP (73.5%), CEF (72.3%), GEN (59%), SXT (53%), CTX (49.4%), and CRO (48.2%) exhibited the highest levels of resistance (Table S10). In contrast, resistance to AMC (3%), AMK (3%), MEM (3%), and CHL (7%) was the lowest (Table S10). Patients with CD had the highest level of resistance to ERY (90%), AMP (80%), GEN (80%), and CIP (66.7%). In contrast, none of the isolates (*n* = 30) displayed resistance to CHL (Figure 4). In patients with UC, 27 isolates (90%) exhibited antimicrobial resistance to AMP whereas resistances to CEF, CIP, and ERY were 83.3, 76.7, and 66.7%, respectively (Figure 4). In patients with IC, all 23 (100%) isolates were resistant to ERY, and 91.3% and 78.3% were resistant to AMP and CIP, respectively (Figure 4). Interestingly, out of 23 isolates from IC patients, none of the isolates showed resistance against three antibiotics, AMC, AMK, and MEM (Figure 4). In HCs, all isolates (50/50, 100%) were resistant to ERY, and the highest levels of resistance were observed against AMP (39/50, 78%), AMK and CEF (32/50, 64%), CIP (29/50, 58%), TET (23/50, 46%), and AMC (22/50, 44%) (Table S11). None of the isolates exhibited resistance to CRO, CTX, and MEM, which indicates that HCs are not ESBL or carbapenemase producers (Figure 4). Of the CRO- or CTX-resistant isolates, 90.7% (39 out of 43) were found to produce ESBL. In patients with CD, UC, and IC, there were 12 (92.3%), 14 (82.4%), and 13 (100%) ESBL-producing isolates, respectively (Figure 6). Additionally, the modified Hodge test demonstrated that none of the strains produced carbapenemase (Figure S6). In contrast, a RAPIDEC^®^ CARBA NP (bioMérieux) test kit confirmed that one in three (33.33%) isolates produced carbapenemase (Table S12).

## 4. Discussion

For all 83 disease-associated isolates, the blast result of 16S rRNA gene top-hit sequences of type-strains showed the highest similarity with *E. fergusonii*. As the 16S rRNA gene sequences alone could not discriminate *E. coli* and *E. fergusonii* correctly, we used matrix-assisted laser desorption/ionization time-of-flight mass spectrometry (MALDI-TOF MS) to identify the strains. The Bruker MALDI Biotyper identification results revealed that most of the strains were *E. coli*. However, some strains remained unidentified, and the identification results for some strains indicated “no peaks found”. Thus, we were unable to correctly identify all the strains as *E. coli* or *E. fergusonii*. This might be due to the lack of a specific identification indicator to detect *E. coli* or *E. fergusonii* in the MALDI Biotyper database. In addition, we attempted to identify the *E. coli* strains using biochemical characterization with API 50CH (bioMérieux) using the APIWEB database (https://apiweb.biomerieux.com; accessed on 3 March 2022), where some of the isolates were identified as *E. coli* and some could not be identified, thereby resulting in an inconclusive identification. In order to correctly identify *E. coli*/*E. fergusonii* in the future, it is recommended that more API test results from *E. coli* or *E. fergusonii* isolates from distinct geographical area be added to the APIWEB database. Finally, we performed a phylogenetic analysis using housekeeping *adk* gene sequences from the *E. coli* MLST scheme to correctly discriminate all *E. coli* strains from *E. fergusonii* strains. Using *adk* gene sequence phylogeny, Maheux et al. previously identified *E. fergusonii* and *Escherichia albertii* [26]. However, we identified four loci (93, 96, 477, and 549) in *adk* gene sequences of *E. coli* and *E. fergusonii* that could correctly discriminate between *E. coli* and *E. fergusonii*, and these loci warrant further research for the accurate identification of *E. coli* and *E. fergusonii* (Table 1 and Figure S5). Notably, five isolates were phylogenetically distant from the *E. fergusonii* and *E. coli* clades (Figure 3). When we individually analysed each *adk* gene sequences in the NCBI database using MegaBLAST or BLASTN, all five isolates displayed the highest similarity to *Citrobacter freundii* (Table S4). In addition, the *adk* gene sequences of these five strains (EB-B-4, EB-C-3, C1-Y-1, C1-E-1, and Y2-B-107) exhibited a different nucleotide arrangement than those of *E. fergusonii* and *E. coli* (Figure S5). This result demonstrates that *adk* gene sequence analysis could also be utilized to distinguish species from another genus, such as *Citrobacter*. However, additional research is required to identify the genetic locus of a specific genus/species. Finally, these findings reveal that *E. coli* and *E. fergusonii* can be accurately distinguished from other members of the genus *Escherichia* by analyzing these four loci in the *adk* gene sequence. Moreover, phylogenetic analysis based on 16S rRNA/*adk* gene sequences also revealed, based on various clusters in the phylogenetic tree, that none of the isolates from disease-associated samples (CD, UC, or IC) or HCs could be distinguished (Figure 1, Figure 2 and Figure 3 and S1–S4).

Additionally, antimicrobial susceptibility profiling revealed a high prevalence of resistance to ERY (88.0%) for disease-associated strains (Table S10). AMR of ERY against CD, UC, IC, and HC isolates was 90%, 66.7%, 100%, and 100%, respectively (Figure 4). Similar to our study, other studies have also reported high ERY resistance (>80%) in *E. coli* strains from human clinical sources in the study of antimicrobial susceptibility patterns of *E. coli* [43]. Likewise, we observed high resistance of disease-associated isolates to first- and third-generation cephalosporins. Overall resistances against CEF (CD, 66.7; UC, 83.3; and IC, 65.2%), CRO (CD, 33.3; UC, 56.7; and IC, 56.5%), and CTX (CD, 40; UC, 53.5; and IC, 56.5%) were 60, 48.2, and 49.4%, respectively (Table S9 and Figure 4). As cephalosporins are essential antibiotics for the treatment of MDR bacterial infections in humans, resistance to these antibiotics may contribute to the emergence of resistance to other cephalosporins, thereby making treatment more challenging [44]. In our study, ≥73% of the isolates were resistant to ciprofloxacin, which is consistent with the 43–82% resistance found in previous studies of *E. coli* associated with CD in the USA and ESBL-producing *Enterobacteriaceae* isolated from patients with IBD in Latvia [14,45]. In a recent study from South Korea, resistance against at least one of the tested antimicrobials in *E. coli* recovered from diarrheal patients was 69.3%, which is much lower than in our study (100%) [46]. Moreover, antibiotic resistance against AMC, AMK, AMP, CIP, CEF, and TET ranged from 44% to 78% among the isolates recovered from HCs (Table S11). These high level of resistance may be attributable to the empirical therapy administered to patients with IBD and IC [6,47]. Importantly, none of the HC group isolates produced ESBL (Figure 5), which indicates that ESBL-producing *E. coli* promotes the pathogenesis of IBD [6] or IC. Skuja et al. observed gut colonization in 65 patients with UC and 100% of the patients with ESBL-producing *E. coli* had a more severe disease than those without gut colonization [48]. Similarly, Meheissen et al. found that >90% of *E coli* isolates from patients with IBD were ESBL-producers [49]. Most ESBL-producing *E. coli* were adherent-invasive *E. coli*, which is directly or indirectly involved in the pathogenesis of IBD (CD or UC) [6,13,14,30,50]. Lastly, the resistance patterns of *E. coli* strains isolated from patients with IBD/IC in different regions and countries may vary, which necessitates the implementation of distinct strategies for preventing the spread of MDR *E. coli* strains in the community and for treating IBD/IC.

This study has some limitations. We analyzed a total of only 83 strains from 11 patients with CD, UC, or IC and 50 strains from 10 healthy South Korean individuals. Owing to the limited sample size and specificity of the populations (only South Korean individuals), the results cannot be generalized to other groups. Furthermore, the isolated *E. coli* strains originated from faeces rather than mucosa. Future research should be focused on ESBL-producing *E. coli* isolates with mucosa-associated origins in larger populations of patients with IBD/IC, with an emphasis on adherent-invasive *E. coli* pathotype in addition to its primary or secondary pathogenicity, to depict the precise role of ESBL-producing *E. coli* in patients with IBD/IC.

In conclusion, our study demonstrated that MLST phylogenetic analysis using *adk* gene sequences could be used to precisely identify *E. coli* or *E. fergusonii*. Four loci in the *adk* gene sequences of *E. coli* from *E. fergusonii* aided in their accurate identification. We observed a frequent and varied occurrence of MDR patterns in antimicrobials commonly used against *E. coli* strains isolated from disease-associated (CD, UC, or IC) Korean individuals. More importantly, these MDR patterns of *E. coli* from CD, UC, and IC isolates can guide the use of appropriate antimicrobials, new therapeutic targets, rational use of antimicrobials, and implementation of administrative guidelines to mitigate the antimicrobial burden in South Korea. In addition, we recommend periodic monitoring of the antimicrobial susceptibility of *E. coli* from human clinical isolates and aim to focus on the role of *E. coli* isolates in IBD or IC etiopathogenesis in future research.

## Figures and Tables

**Table 1:** Differentiating *adk* gene nucleotides of *E. coli* against *E. fergusonii* strains.

Loci	*adk* Gene Sequence Nucleotide	
	*E. coli*	*E. fergusonii*
93	T	G
96	C	T
477	T	C
549	C	A

## Data Availability

All the 16S rRNA gene sequences and *adk* gene sequences have been deposited in the public database (GenBank/EMBL/DDBJ).

## Supplementary Materials

The following supporting information can be downloaded at https://www.mdpi.com/article/10.3390/antibiotics12010154/s1: Figure S1: The neighbour-joining phylogenetic tree based on 16S rRNA gene sequences of isolated *E. coli*/*E. fergusonii* strains from patients with IBD and HCs; Figure S2: The neighbour-joining phylogenetic tree based on 16S rRNA gene sequences of isolated *E. coli*/*E. fergusonii* strains from patients with CD and HCs; Figure S3: The neighbour-joining phylogenetic tree based on 16S rRNA gene sequences of isolated *E. coli*/*E. fergusonii* strains from patients with UC and HCs; Figure S4: The neighbour-joining phylogenetic tree based on 16S rRNA gene sequences of isolated *E. coli*/*E. fergusonii* strains from patients with IC and HCs; Figure S5: Differential nucleotides of *E. coli* and *E. fergusonii* with respective loci; Figure S6: Modified Hodge test to confirm carbapenemase producers; Table S1: List of culture media. Table S2: Species identification based on 16S rRNA gene sequences of isolated strains from disease-associated (IBD/IC) faecal samples; Table S3: Species identification based on 16S rRNA gene sequences of isolated strains from faecal samples of HCs; Table S4: Species identification based on a*dk* gene sequences of isolated strains from disease-associated (IBD/IC) faecal samples; Table S5: Resistance patterns of 83 strains isolated from disease-associated (CD, UC, and IC) Korean patients. Table S6: Resistance patterns of 50 strains isolated from HCs; Table S7: Resistance patterns of 30 strains isolated from faecal sample with CD; Table S8: Resistance patterns in 30 strains isolated from faecal sample with UC; Table S9: Resistance patterns of 23 strains isolated from faecal sample with IC; Table S10: Antimicrobial susceptibility of 83 isolates from disease-associated (IBD/IC) Korean individuals; Table S11: Antimicrobial susceptibility of 50 isolates from HCs; Table S12: RAPIDEC CARBA NC test for carbapenemase producers.

## Abbreviations

IBD: Inflammatory bowel disease; IC: Ischemic colitis; AMR: Antimicrobial resistance; MDR: Multidrug resistance; ESBL: Extended-spectrum beta-lactamase; MIC: Minimum inhibitory concentration; AMC: Amoxicillin-clavulanic acid; AMK: Amikacin; AMP: Ampicillin; CIP: Ciprofloxacin; CHL: Chloramphenicol; CST: Colistin; CEF: Cephalothin; CRO: Ceftriaxone; CTX: Cefotaxime; ERY: Erythromycin; GEN: Gentamicin; MEM: Meropenem; SXT: Trimethoprim-sulfamethoxazole; TET: Tetracycline.
