# Supplementary material for: Differentiation of Escherichia fergusonii and Escherichia coli Isolated from Patients with Inflammatory Bowel Disease/Ischemic Colitis and Their Antimicrobial Susceptibility Patterns
Source: Antibiotics (Basel). 2023 Jan 11;12(1):154. doi: 10.3390/antibiotics12010154 (PMC9854958; doi:10.3390/antibiotics12010154)
Supplement: Supplementary file 1 [file antibiotics-12-00154-s001.zip › antibiotics-2088653-Supplementary materials.pdf]

Supplementary Materials

Differentiation of *Escherichia fergusonii* and *Escherichia coli* Isolated from Patients with Inflammatory Bowel Disease/ischemic Colitis and Their Antimicrobial Susceptibility Patterns

Ram Hari Dahal, Yoon-Jung Choi, Shukho Kim and Jungmin Kim *

Department of Microbiology, School of Medicine, Kyungpook National University, Daegu 41944, Republic of Korea

* Correspondence: minkim@knu.ac.kr; Tel.: +82-53-420-4840

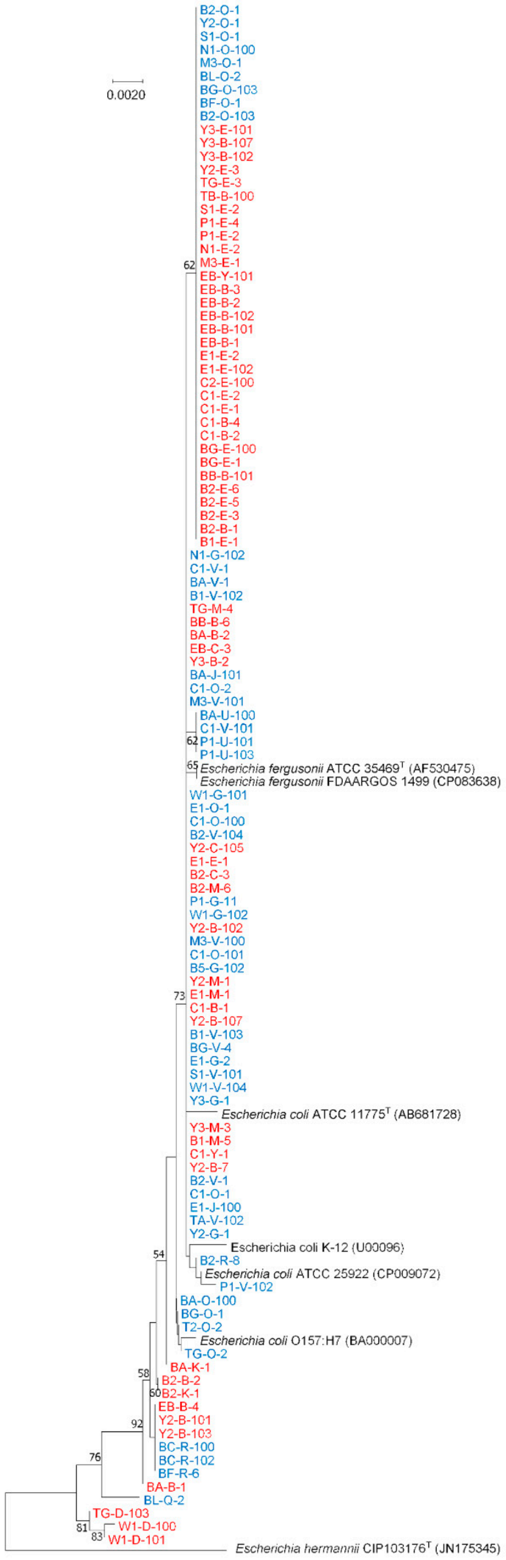

Figure S1. The neighbour-joining phylogenetic tree based on 16S rRNA gene sequences of isolated *E. coli*/*E. fergusonii* strains from patients with IBD and healthy controls (HCs). The numbers at the nodes indicate the percentage of 1000 bootstrap replicates. *Escherichia hermannii* CIP 103176^{T} was used an outgroup. The accession numbers of retrieved 16S rRNA gene sequences are given in parentheses. Strains name with colour codes (red = IBD; blue = HCs).

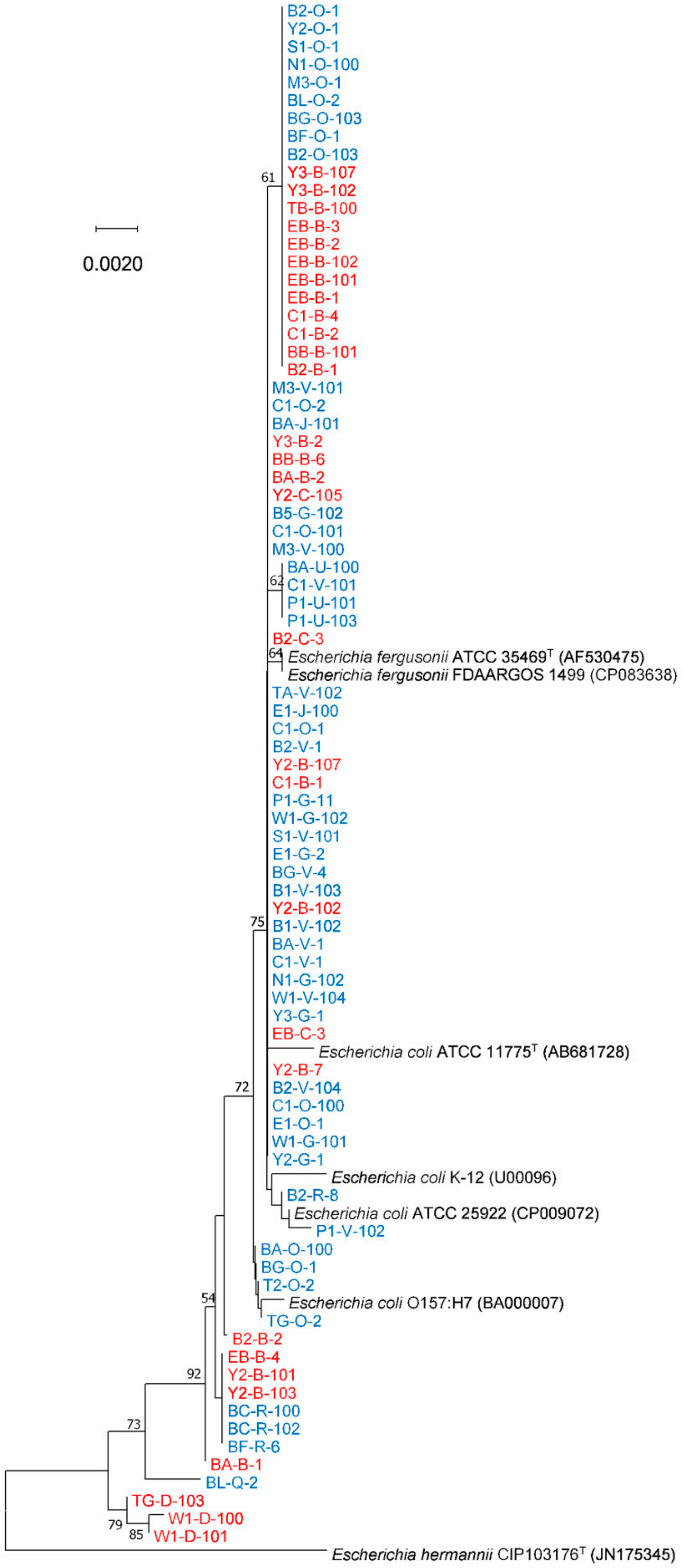

Figure S2. The neighbour-joining phylogenetic tree based on 16S rRNA gene sequences of isolated *E. coli*/*E. fergusonii* strains from patients with CD and healthy controls (HCs). The numbers at the nodes indicate the percentage of 1000 bootstrap replicates. *Escherichia hermannii* CIP 103176^{T} was used an outgroup. The accession numbers of retrieved 16S rRNA gene sequences are given in parentheses. Strains name with colour codes (red = CD; blue = HCs).

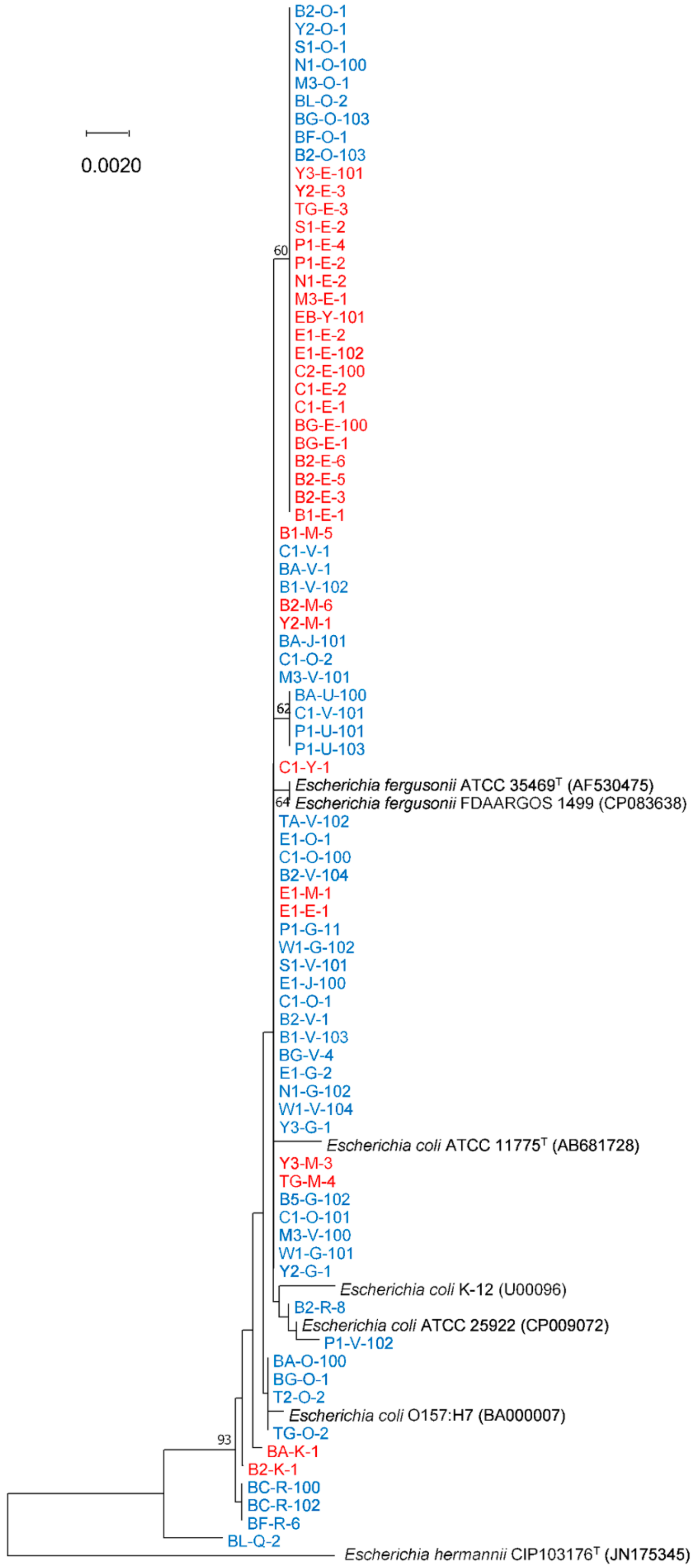

Figure S3. The neighbour-joining phylogenetic tree based on 16S rRNA gene sequences of isolated *E. coli*/*E. fergusonii* strains from patients with UC and healthy controls (HCs). The numbers at the nodes indicate the percentage of 1000 bootstrap replicates. *Escherichia hermannii* CIP 103176^{T} was used an outgroup. The accession numbers of retrieved 16S rRNA gene sequences are given in parentheses. Strains name with colour codes (red = UC; blue = HCs).

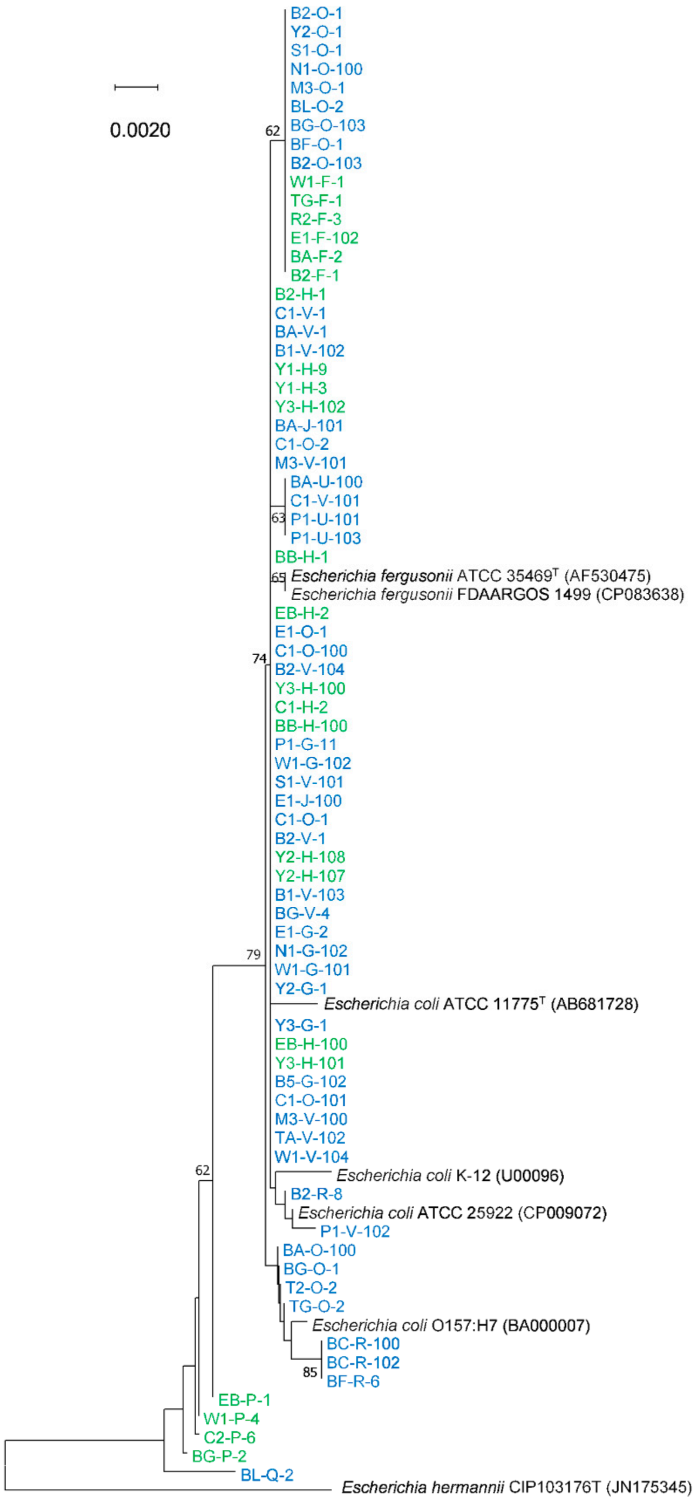

Figure S4. The neighbour-joining phylogenetic tree based on 16S rRNA gene sequences of isolated *E. coli*/*E. fergusonii* strains from patients with IC and healthy controls (HCs). The numbers at the nodes indicate the percentage of 1000 bootstrap replicates. *Escherichia hermannii* CIP 103176^T was used an outgroup. The accession numbers of retrieved 16S rRNA gene sequences are given in parentheses. Strains name with colour codes (green = IC; blue = HCs).

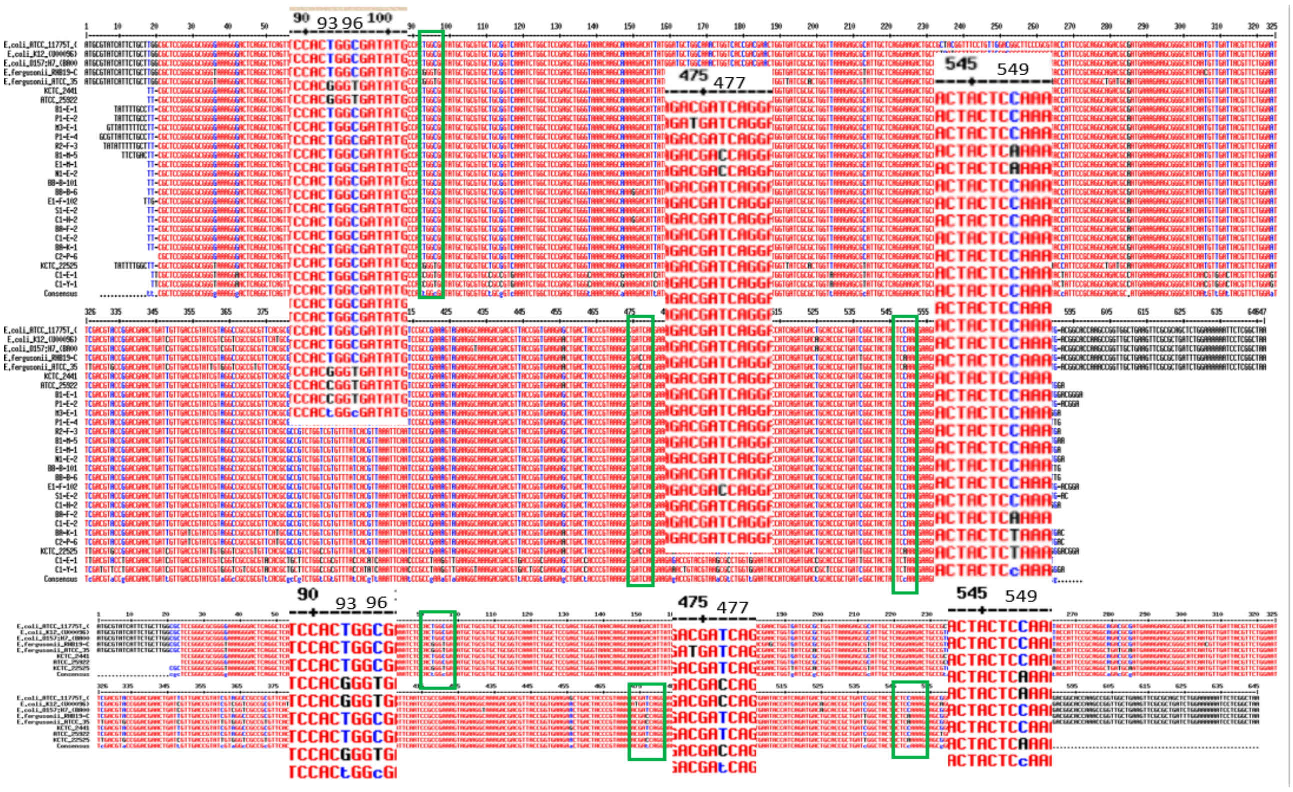

Figure S5. Differential nucleotides of *E. coli* and *E. fergusonii* with respective loci.

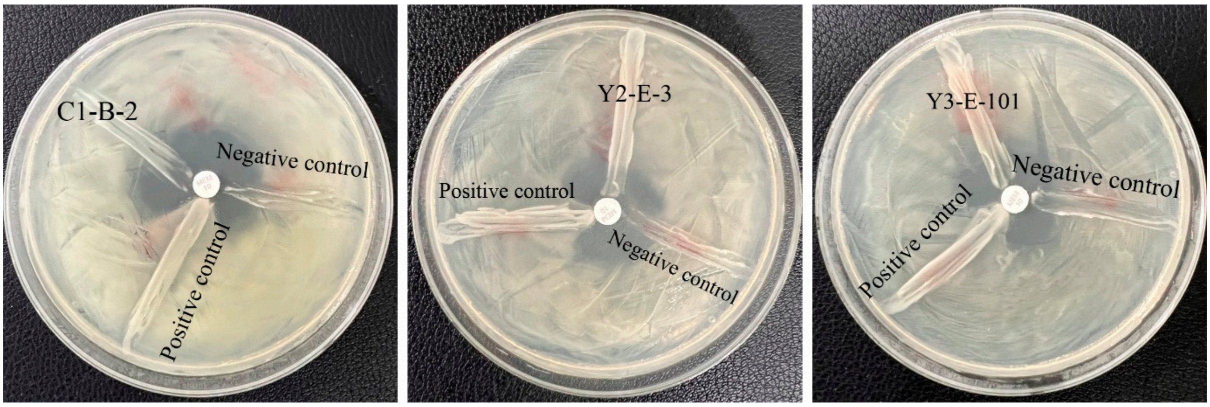

Figure S6. Modified Hodge test to confirm carbapenemase producers. Positive control: *Klebsiella pneumonie* KNU 115; negative control: *Pseudomonas aeruginosa* ATCC 27853; test strains: meropenem resistant strains (C1-B-2, Y2-E-3, and Y3-E-101).

Table S5. Resistance patterns of 83 strains isolated from disease associated (CD, UC, and IC) Korean patients.

No. of Antibiotics	No. of Isolates (%)	Resistance Pattern (No. of Isolates)
1	1 (1.2)	ERY (*n* = 1)
2	4 (4.8)	AMK, ERY (*n* = 1)
		AMP, GEN (*n* = 1)
		CIP, TET (*n* = 1)
		ERY, SXT (*n* = 1)
3	6 (7.2)	AMP, CEF, ERY (*n* = 1)
		AMP, CEF, SXT (*n* = 2)
		CST, CEF, ERY (*n* = 1)
		CRO, CTX, ERY (*n* = 1)
		ERY, SXT, TET (*n* = 1)
4	6 (7.2)	AMP, CIP, CST, ERY (*n* = 1)
		AMP, CIP, ERY, GEN (*n* = 2)

		AMP, ERY, SXT, TET (*n* = 1)
		CIP, CST, ERY, TET (*n* = 1)
		CIP, CRO, CTX, ERY (*n* = 1)
5	16 (19.3)	AMK, CIP, CST, CEF, MEM (*n* = 1)
		AMP, CIP, CHL, ERY, GEN (*n* = 1)
		AMP, CIP, CEF, ERY, GEN (*n* = 5)
		AMP, CIP, CRO, CTX, ERY (*n* = 1)
		AMP, CIP, CRO, ERY, GEN (*n* = 1)
		AMP, CIP, ERY, GEN, TET (*n* = 1)
		AMP, CIP, ERY, SXT, TET (*n* = 1)
		AMP, CST, ERY, SXT, TET (*n* = 1)
		AMP, CEF, CRO, CTX, ERY (*n* = 1)
		AMP, CEF, ERY, GEN, TET (*n* = 1)
		CIP, CHL, CST, ERY, TET (*n* = 1)
		CEF, ERY, GEN, SXT, TET (*n* = 1)
6	8 (9.6)	AMC, AMP, CEF, GEN, SXT, TET (*n* = 1)
		AMP, CIP, CST, CEF, ERY, SXT (*n* = 1)
		AMP, CIP, CST, ERY, SXT, TET (*n* = 1)
		AMP, CIP, CEF, CRO, CTX, ERY (*n* = 3)
		AMP, CIP, CTX, ERY, GEN, SXT (*n* = 1)
		AMP, CEF, ERY, GEN, SXT, TET (*n* = 1)
7	20 (24.1)	AMC, AMP, CEF, ERY, GEN, SXT, TET (*n* = 1)
		AMP, CIP, CHL, CEF, CRO, CTX, ERY (*n* = 1)
		AMP, CIP, CST, CEF, CRO, CTX, ERY (*n* = 2)
		AMP, CIP, CEF, CRO, CTX, ERY, GEN (*n* = 3)
		AMP, CIP, CEF, CRO, CTX, ERY, SXT (*n* = 3)
		AMP, CIP, CEF, ERY, GEN, SXT, TET (*n* = 4)
		AMP, CIP, CRO, CTX, ERY, SXT, TET (*n* = 1)
		AMP, CST, CEF, ERY, GEN, SXT, TET (*n* = 2)
		AMP, CEF, CRO, CTX, ERY, GEN, SXT (*n* = 1)
		AMP, CEF, CRO, CTX, GEN, SXT, TET (*n* = 1)
		AMP, CEF, CRO, ERY, GEN, SXT, TET (*n* = 1)
8	12 (14.5)	AMP, CIP, CHL, CEF, CRO, CTX, ERY, GEN (*n* = 2)
		AMP, CIP, CHL, CEF, CRO, CTX, ERY, SXT (*n* = 1)
		AMP, CIP, CST, CEF, CRO, CTX, ERY, GEN (*n* = 1)
		AMP, CIP, CST, CEF, ERY, GEN, SXT, TET (*n* = 1)
		AMP, CIP, CEF, CRO, CTX, ERY, SXT, TET (*n* = 2)
		AMP, CIP, CEF, CRO, CTX, GEN, SXT, TET (*n* = 1)
		AMP, CIP, CEF, CTX, ERY, GEN, SXT, TET (*n* = 3)
		AMP, CEF, CRO, CTX, ERY, GEN, SXT, TET (*n* =1)
9	8 (9.6)	AMC, AMP, CIP, CEF, CRO, CTX, ERY, GEN, SXT (*n* = 1)
		AMP, CIP, CST, CEF, CRO, ERY, GEN, SXT, TET (*n* = 1)
		AMP, CIP, CEF, CRO, CTX, ERY, GEN, SXT, TET (*n* = 5)
		AMP, CIP, CEF, CTX, ERY, GEN, MEM, SXT, TET (*n* = 1)
10	2 (2.4)	AMK, AMP, CIP, CST, CEF, CRO, CTX, ERY, GEN, MEM (*n* = 1)
		AMP, CIP, CHL, CEF, CRO, CTX, ERY, GEN, SXT, TET (*n* = 1)
**Total**	**83 strains**	**58 patterns**

Table S6. Resistance patterns of 50 strains isolated from healthy controls (HCs).

No. of Antibiotics	No. of Isolates (%)	Resistance Pattern (No. of Isolates)
1	3 (6.0)	ERM (*n* = 3)
2	2 (4.0)	AMK, ERM (*n* = 1)
		CPH, ERM (*n* = 1)
3	4 (8.0)	AMP, COL, ERM (*n* = 1)
		CIP, CPH, ERM *(n* = 2)
		COL, CPH, ERM (*n* = 1)
4	7 (14.0)	AMC, AMK, AMP, ERM (*n* = 1)
		AMK, AMP, COL, ERM (*n* = 1)
		AMK, AMP, ERM, TET (*n* = 1)
		AMK, CIP, CPH, ERM (*n* = 2)

		AMP, CIP, CPH, ERM (*n* = 1)
		AMP, ERM, SXT, TET (*n* = 1)
5	10 (20.0)	AMC, AMK, AMP, CIP, ERM (*n* = 2)
		AMC, AMK, AMP, CPH, ERM (*n* = 1)
		AMK, AMP, CIP, ERM, TET (*n* = 2)
		AMK, AMP, COL, ERM, TET (*n* = 1)
		AMK, AMP, CPH, ERM, TET (*n* = 1)
		AMP, CIP, CHL, CPH, ERM (*n* = 1)
		AMP, CIP, CPH, ERM, TET (*n* = 1)
		CIP, CHL, COL CPH, ERM (*n* = 1)
6	11 (22.0)	AMC, AMK, AMP, CIP, CPH, ERM (*n* = 1)
		AMC, AMK, AMP, CHL, CPH, ERM (*n* = 2)
		AMC, AMK, AMP, CHL, ERM, TET (*n* = 1)
		AMC, AMK, AMP, ERM, SXT, TET (*n* = 1)
		AMC, AMP, CIP, CHL, CPH, ERM (*n* = 1)
		AMC, AMP, CIP, CPH, ERM, TET (*n* = 1)
		AMK, AMP, CIP, COL, ERM, TET (*n* = 1)
		AMK, AMP, CIP, CPH, ERM, SXT (*n* = 1)
		AMP, CIP, CPH, ERM, GEN, TET (*n* = 1)
		AMP, CIP, CPH, ERM, SXT, TET (*n* = 1)
7	8 (16.0)	AMC, AMK, AMP, CIP, CHL, CPH, ERM (*n* = 1)
		AMC, AMK, AMP, CIP, COL, CPH, ERM (*n* = 1)
		AMC, AMK, AMP, CIP, CPH, ERM, TET (*n* = 2)
		AMC, AMK, AMP, CHL, COL, CPH, ERM (*n* = 1)
		AMC, AMK, AMP, COL, ERM, SXT, TET (*n* = 1)
		AMC, AMP, CIP, COL, CPH, ERM, TET (*n* = 1)
		AMK, AMP, CIP, COL, CPH, ERM, TET (*n* = 1)
8	3 (6.0)	AMC, AMK, AMP, CIP, CPH, ERM, SXT, TET (*n* = 2)
		AMK, AMP, CIP, COL, CPH, ERM, GEN, TET (*n* = 1)
9	2 (4.0)	AMC, AMK, AMP, CIP, COL, CPH, ERM, GEN, TET (*n* = 1)
		AMK, AMP, CIP, COL, CPH, ERM, GEN, SXT, TET (*n* = 1)
**Total**	**50 strains**	**41 patterns**

Table S7. Resistance patterns of 30 strains isolated from faecal sample with Chron's disease (CD).

No. of Antibiotics	No. of Isolates (%)	Resistance Pattern (No. of Isolates)
1	1 (3.3)	ERY (*n* = 1)
2	1 (3.3)	AMK, ERY (*n* = 1)
3	2 (6.7)	CST, CEF, ERY (*n* = 1)
		CRO, CTX, ERY (*n* = 1)
4	4 (13.3)	AMP, CIP, CST, ERY (*n* = 1)
		AMP, CIP, ERY, GEN (*n* = 1)
		CIP, CST, ERY, TET (*n* = 1)
		CIP, CRO, CTX, ERY (*n* = 1)
5	3 (10)	AMP, CIP, CEF, ERY, GEN (*n* = 1)
		AMP, CIP, CRO, ERY, GEN (*n* = 1)
		AMP, CIP, ERY, GEN, TET (*n* = 1)
6	3 (10)	AMC, AMP, CEF, GEN, SXT, TET (*n* = 1)
		AMP, CIP, CTX, ERY, GEN, SXT (*n* = 1)
		AMP, CEF, ERY, GEN, SXT, TET (*n* = 1)
7	9 (30)	AMC, AMP, CEF, ERY, GEN, SXT, TET (*n* = 1)
		AMP, CIP, CEF, CRO, CTX, ERY, GEN (*n* = 1)
		AMP, CIP, CEF, ERY, GEN, SXT, TET (*n* = 4)
		AMP, CST, CEF, ERY, GEN, SXT, TET (*n* = 1)
		AMP, CEF, CRO, CTX, ERY, GEN, SXT (*n* = 1)
		AMP, CEF, CRO, ERY, GEN, SXT, TET (*n* = 1)
8	5 (16.7)	AMP, CIP, CST, CEF, CRO, CTX, ERY, GEN (*n* = 1)
		AMP, CIP, CST, CEF, ERY, GEN, SXT, TET (*n* = 1)
		AMP, CIP, CEF, CRO, CTX, GEN, SXT, TET (*n* = 1)
		AMP, CIP, CEF, CTX, ERY, GEN, SXT, TET (*n* = 1)
		AMP, CEF, CRO, CTX, ERY, GEN, SXT, TET (*n* = 1)
9	2 (6.7)	AMP, CIP, CEF, CRO, CTX, ERY, GEN, SXT, TET (*n* = 1)
		AMP, CIP, CEF, CTX, ERY, GEN, MEM, SXT, TET (*n* = 1)
**Total**	**30 strains**	**27 patterns**

Table S8. Resistance patterns of 30 strains isolated from faecal sample with ulcerative colitis (UC).

No. of Antibiotics	No. of Isolates (%)	Resistance Pattern (No. of Isolates)
2	2 (6.7)	CIP, TET (*n* = 1)
		AMP, GEN (*n* = 1)
3	3 (10)	AMP, CEF, ERY (*n* = 1)
		AMP, CEF, SXT (*n* = 2)
4	1 (3.3)	AMP, CIP, ERY, GEN (*n* = 1)
5	8 (26.7)	AMK, CIP, CST, CEF, MEM (*n* = 1)
		AMP, CIP, CHL, ERY, GEN (*n* = 1)
		AMP, CIP, CEF, ERY, GEN (*n* = 3)
		AMP, CEF, CRO, CTX, ERY (*n* = 1)
		AMP, CEF, ERY, GEN, TET (*n* = 1)
		CIP, CHL, CST, ERY, TET (*n* = 1)
7	5 (16.7)	AMP, CIP, CHL, CEF, CRO, CTX, ERY (*n* = 1)
		AMP, CIP, CST, CEF, CRO, CTX, ERY (*n* = 1)
		AMP, CIP, CEF, CRO, CTX, ERY, SXT (*n* = 2)
		AMP, CEF, CRO, CTX, GEN, SXT, TET (*n* = 1)
8	3 (10)	AMP, CIP, CEF, CRO, CTX, ERY, SXT, TET (*n* = 1)
		AMP, CIP, CEF, CRO, CTX, GEN, SXT, TET (*n* = 2)
9	6 (20)	AMC, AMP, CIP, CEF, CRO, CTX, ERY, GEN, SXT (*n* = 1)
		AMP, CIP, CST, CEF, CRO, ERY, GEN, SXT, TET (*n* = 1)
		AMP, CIP, CEF, CRO, CTX, ERY, GEN, SXT, TET (*n* = 4)
10	2 (6.7)	AMK, AMP, CIP, CST, CEF, CRO, CTX, ERY, GEN, MEM (*n* = 1)
		AMP, CIP, CHL, CEF, CRO, CTX, ERY, GEN, SXT, TET (*n* = 1)
**Total**	**30 strains**	**22 patterns**

Table S9. Resistance patterns of 23 strains isolated from faecal sample with ischemic colitis (IC).

No. of Antibiotics	No. of Isolates (%)	Resistance Pattern (No. of Isolates)
2	1 (4.3)	ERY, SXT (*n* = 1)
3	1 (4.3)	ERY, SXT, TET (*n* = 1)
4	1 (4.3)	AMP, ERY, SXT, TET (*n* = 1)
5	5 (21.7)	AMP, CIP, CEF, ERY, GEN (*n* = 1)
		AMP, CIP, CRO, CTX, ERY (*n* = 1)
		AMP, CIP, ERY, SXT, TET (*n* = 1)
		AMP, CST, ERY, SXT, TET (*n* = 1)
		CEF, ERY, GEN, SXT, TET (*n* = 1)
6	5 (21.7)	AMP, CIP, CST, CEF, ERY, SXT (*n* = 1)
		AMP, CIP, CST, ERY, SXT, TET (*n* = 1)
		AMP, CIP, CEF, CRO, CTX, ERY (*n* = 3)
7	6 (26.1)	AMP, CIP, CST, CEF, CRO, CTX, ERY (*n* = 1)
		AMP, CIP, CEF, CRO, CTX, ERY, GEN (*n* = 2)
		AMP, CIP, CEF, CRO, CTX, ERY, SXT (*n* = 1)
		AMP, CIP, CRO, CTX, ERY, SXT, TET (*n* = 1)
		AMP, CST, CEF, ERY, GEN, SXT, TET (*n* = 1)
8	4 (17.4)	AMP, CIP, CHL, CEF, CRO, CTX, ERY, GEN (*n* = 2)
		AMP, CIP, CHL, CEF, CRO, CTX, ERY, SXT (*n* = 1)
		AMP, CIP, CEF, CRO, CTX, ERY, SXT, TET (*n* = 1)
**Total**	**23 strains**	**19 patterns**

Table S10. Antimicrobial susceptibility of 83 isolates from disease-associated (CD, UC, or IC) Korean individuals.

Antimicrobials	AMC	AMK	AMP	CIP	CHL	CST	CEF	CRO	CTX	ERY	GEN	MEM	SXT	TET
Resistant	3 (3.6%)	3 (3.6%)	72 (86.7%)	61 (73.5%)	7 (8.4%)	17 (20.5%)	60 (72.3%)	40 (48.2%)	41 (49.4%)	73 (88.0%)	49 (59%)	3 (3.6%)	44 (53%)	40 (48.2%)
Intermediate	37 (44.6%)	13 (15.7%)	0 (0%)	0 (0%)	20 (24.1%)	0 (0%)	11 (13.3%)	1 (1.2%)	0 (0%)	1 (1.2%)	1 (1.2%)	0 (0%)	0 (0%)	7 (8.4%)
Susceptible	43 (51.8%)	67 (80.7%)	11 (13.3%)	22 (26.5%)	56 (67.5%)	66 (79.5%)	12 (14.5%)	42 (50.6%)	42 (50.6%)	9 (10.8%)	33 (39.8%)	80 (96.4%)	39 (47%)	36 (43.4%)

Table S11. Antimicrobial susceptibility of 50 isolates from healthy controls (HCs).

Antimicrobials	AMC	AMK	AMP	CIP	CHL	CST	CEF	CRO	CTX	ERY	GEN	MEM	SXT	TET
Resistant	22 (44%)	32 (64%)	39 (78%)	29 (58%)	8 (16%)	14 (28%)	32 (64%)	0 (0%)	0 (0%)	50 (100%)	5 (10%)	0 (0%)	8 (16%)	23 (46%)
Intermediate	17 (34%)	5 (10%)	1 (2%)	0 (0%)	26 (52%)	0 (0%)	15 (30%)	0 (0%)	0 (0%)	0 (0%)	14 (28%)	0 (0%)	0 (0%)	7 (14%)
Susceptible	11 (22%)	13 (26%)	10 (20%)	21 (42%)	16 (32%)	36 (72%)	3 (6%)	50 (100%)	50 (100%)	0 (0%)	31 (62%)	50 (100%)	42 (84%)	20 (40%)

Table S12. RAPIDEC CARBA NC test for carbapenemase producers.

SN	Strains	RAPIDEC CARBA NC		
		**d**	**e**	**Result**
**1**	C1-B-2	Red	Orange	+
2	Y2-E-3	Red	Red	-
3	Y3-E-101	Red	Red	-
4	*Klebsiella pneumoniae* KNU 1115	Red	Yellow	+
5	*Pseudomonas aeruginosa* ATCC 27853	Orange	Orange	-
